# Operationalisation of a standardised scoring system to assess adherence to the World Cancer Research Fund/American Institute for Cancer Research cancer prevention recommendations in the UK biobank

**DOI:** 10.3389/fnut.2023.1011786

**Published:** 2023-02-10

**Authors:** Fiona C. Malcomson, Solange Parra-Soto, Liya Lu, Frederick K. Ho, Aurora Perez-Cornago, Marissa M. Shams-White, Moniek van Zutphen, Ellen Kampman, Renate M. Winkels, Panagiota Mitrou, Martin Wiseman, Dora Romaguera, Carlos Celis-Morales, Linda Sharp, John C. Mathers

**Affiliations:** ^1^Human Nutrition and Exercise Research Centre, Centre for Healthier Lives, Population Health Sciences Institute, Newcastle University, Newcastle upon Tyne, United Kingdom; ^2^School of Health and Wellbeing, University of Glasgow, Glasgow, United Kingdom; ^3^School of Cardiovascular and Metabolic Health, University of Glasgow, Glasgow, United Kingdom; ^4^Centre for Cancer, Population Health Sciences Institute, Newcastle University, Newcastle upon Tyne, United Kingdom; ^5^Cancer Epidemiology Unit, Nuffield Department of Population Health, University of Oxford, Oxford, United Kingdom; ^6^Risk Factor Assessment Branch, Epidemiology and Genomics Research Program, Division of Cancer Control and Population Sciences, National Cancer Institute, Bethesda, MD, United States; ^7^Division of Human Nutrition and Health, Wageningen University and Research, Wageningen, Netherlands; ^8^Radboud Institute for Health Sciences, Radboud University Medical Center, Nijmegen, Netherlands; ^9^World Cancer Research Fund International, London, United Kingdom; ^10^Health Research Institute of the Balearic Islands (IdISBa), Palma de Mallorca, Spain; ^11^CIBER Physiopathology of Obesity and Nutrition (CIBEROBN), Madrid, Spain; ^12^Human Performance Lab, Education, Physical Activity and Health Research Unit, University Católica del Maule, Talca, Chile

**Keywords:** cancer prevention recommendations, lifestyle, scoring system, cancer risk, lifestyle recommendations

## Abstract

**Introduction:**

In 2018, The World Cancer Research Fund (WCRF)/American Institute for Cancer Research (AICR) published ten evidence-based Cancer Prevention Recommendations designed to reduce the risk of cancer *via* improved lifestyle behaviours. In 2019, Shams-White and colleagues created the “2018 WCRF/AICR Score” which aimed to standardise how adherence to these recommendations is assessed. The standardised scoring system includes seven of the recommendations concerning weight, physical activity and diet, with an optional eighth recommendation on breastfeeding. To promote transparency and reproducibility, the present paper describes the methodology for operationalisation of the standardised scoring system in the UK Biobank.

**Methods:**

UK Biobank recruited >500,000 individuals aged 37–73 years, between 2006 and 2010. In 2021, we held a workshop with experts which aimed to reach consensus on how to operationalise the scoring system using data available within UK Biobank. We used data on anthropometric measurements, physical activity and diet to calculate adherence scores. 24 h dietary assessment data were used to measure adherence to the following recommendations: “Eat a diet rich in wholegrains, vegetables, fruit, and beans”, “Limit consumption of “fast foods” and other processed foods high in fat, starches or sugars” and “Limit consumption of sugar-sweetened drinks”; food frequency questionnaire data were used to assess adherence to “Limit consumption of red and processed meat” and “Limit alcohol consumption”. Participants were allocated points for meeting, partially meeting or not meeting each recommendation, using cut-offs defined in the standardised scoring system.

**Results:**

At our workshop, discussions included the use of national guidelines to assess adherence to the recommendation on alcohol consumption, as well as challenges faced including defining the adapted ultra-processed food variables. A total score was calculated for 158,415 participants (mean 3.9 points, range 0–7 points). We also describe the methodology to derive a partial 5-point adherence score using data from the food frequency questionnaire in 314,616 participants.

**Conclusion:**

We describe the methodology used to estimate adherence to the 2018 WCRF/AICR Cancer Prevention Recommendations for participants in the UK Biobank, including some of the challenges faced operationalising the standardised scoring system.

## 1. Introduction

In 2018, the World Cancer Research Fund (WCRF)/American Institute for Cancer Research (AICR) published ten updated, evidence-based Cancer Prevention Recommendations designed to reduce the risk of cancer, *via* modifiable lifestyle behaviours including promoting healthier diets and physical activity (1). In 2019, Shams-White and colleagues created a scoring system to standardise how adherence to these Cancer Prevention Recommendations is assessed and to provide a framework to improve consistency and comparability across studies (2). The standardised scoring system includes seven of the ten 2018 WCRF/ACIR Cancer Prevention Recommendations concerning weight, physical activity and diet, with an optional eighth recommendation on breastfeeding, and is calculated for individuals. The score creators excluded the recommendation to avoid dietary supplements for cancer prevention and consume nutrients through food alone as this is largely addressed through the other five dietary recommendations, and the recommendation specific to cancer survivors as adherence to this would be derived from a composite measure of the other score components (2). Each recommendation is worth a maximum 1 point for full adherence, half a point for partially meeting the recommendation, and 0 points for not meeting the recommendation, yielding a maximum score of 7 points (8 if the optional recommendation is included).

The standardised scoring system used to assess adherence to the Cancer Prevention Recommendations has been applied, at least in part, in several studies, in countries including The Netherlands (3), Australia (4), United States (5, 6), Spain (7, 8), Italy and Switzerland (9). However, to our knowledge, it has not previously been fully applied in a UK cohort. It is important to assess adherence to lifestyle recommendations and to operationalise such scoring systems across different countries and studies because of the differences in eating patterns, lifestyle and study methods. In the Cancer Lifestyle Prevention Recommendations (CALIPER) UK Study, we aim to investigate relationships between adherence to the Cancer Prevention Recommendations and cancer risk and survival using data from the UK Biobank Study, a prospective cohort study, which recruited over half a million participants across the UK.

The collection of diet and nutrition information presents many challenges, including the selection of the most appropriate method to obtain the highest quality data possible whilst considering the purpose of the data collection and participant burden. The UK Biobank assessed dietary intake using two methods: a touchscreen questionnaire asking 29 diet-related questions (similar to a food-frequency questionnaire (FFQ)) and, in over 200,000 participants, used a web-based 24 h dietary assessment tool “Oxford WebQ” to collect more detailed information (10). A further complexity of the dietary data available within the UK Biobank is that, at the end of the recruitment period, participants were invited to complete another web-based dietary assessment on four occasions between February 2011 and June 2012. Thus, the number of dietary assessments completed by each participant, as well as the dates when these were completed, vary.

Therefore, the aim of this paper is to describe the methodology used to operationalise the standardised scoring system in the UK Biobank, to promote transparency and reproducibility, as encouraged by Shams-White and colleagues (2). We also describe the methodology applied to derive a partial, modified 5-point adherence score using data from the FFQ, for which we have data for a greater number of UK Biobank participants.

## 2. Materials and equipment

### 2.1. The UK Biobank study

The UK Biobank is a prospective cohort study which recruited 503,317 individuals from the general population aged 37–73 years, 56% female, from 22 recruitment (henceforth “assessment”) centres across the UK (England, Scotland and Wales) between 2006 and 2010. Full eligibility criteria and recruitment and follow-up methods for UK Biobank are reported on the UK Biobank website (11). The UK Biobank was conducted in accordance with the Declaration of Helsinki and was approved by the North West Multi-Centre Research Ethics Committee (REC reference: 12/NW/03820). At the baseline study visit at an assessment centre, a touchscreen questionnaire was used to collect data on sociodemographic factors, diet and general health, and other participant characteristics, blood samples were collected, and anthropometric measurements were taken, as described below.

### 2.2. Dietary assessment within the UK Biobank

Two methods of dietary assessment were used within the UK Biobank during different periods of recruitment. Initially, a short FFQ-based approach, including 29 questions on diet and 18 on alcohol, formed part of the baseline touchscreen questionnaire and was completed by all participants at the assessment centre. The questionnaire captured information on the frequency of consumption of major food groups, including fruits and vegetables, fish, meat and cheese, in the last year.

Those participants that were recruited towards the end of the recruitment period (between 2009 and September 2010), also completed a 24 h dietary assessment, the Oxford WebQ (12), which captures information on up to 206 food and 32 drink items. In addition, between February 2011 and June 2012, there were 4 cycles, separated by 3–4 months, in which all participants who had provided a valid email address were invited to complete the 24 h dietary assessment at home. In total, 210,128 participants (42% of the total cohort) completed at least one 24 h dietary assessment and 126,096 (25% of the total cohort) completed at least two (10). Further details about the dietary assessments, including reproducibility and agreement between the two methods have been published (10). There was moderate to substantial agreement in the responses to the main food groups at baseline and approximately 4 years later in 20,348 participants, with κ Coefficients with quadratic weighting between 0.52 (for dried fruit intake) and 0.71 (for poultry intake) (κ values between 0.61–0.80 and between 0.41–0.60 represent substantial and moderate agreement, respectively) (10). Furthermore, there was reliable ranking of participants for all foods and food groups according to the touchscreen questionnaire categorisation when compared with group mean intakes from the 24 h dietary assessment (10).

In the present study, we used data from the 24 h dietary assessment (Oxford WebQ) for intakes of food groups for which there is not much variation from day to day, to assess adherence to the recommendations on the intakes of fruits and vegetables, dietary fibre, ultra-processed foods and sugar-sweetened drinks. We used FFQ data to capture the usual intake of foods not consumed daily, for operationalisation of the red meat and alcohol recommendations where the cut-offs are described as intake “per week”, so as not to over or underestimate consumption of these foods.

### 2.3. Processing of 24 h dietary assessment data

For operationalisation of the recommendations using 24 h dietary assessment data, intakes were derived by taking the mean of the completed assessments. We excluded any assessments for which the participant answered “no” to the following question “Would you say that what you ate and drank yesterday was fairly typical for you? (UK Biobank data-field 100020). We also excluded any dietary assessments with extreme energy intakes (based on the “Estimated Nutrients” data-field 100002), using the cut-offs described by Perez-Cornago et al. (13); < 3,347 or > 17,573 kJ per day (< 800 or > 4,200 kcal/per day) for men and < 2092 or > 14,644 kJ per day (< 600 or > 3,500 kcal per day) for women. Perez-Cornago et al. (13) advise that at least two 24 h dietary assessments are used, if possible, when investigating diet-disease associations, as a single dietary assessment is unlikely to reflect habitual intakes, and we will apply this for our future diet-cancer analyses.

We used the updated portion sizes assigned by Perez-Cornago et al. (13) and, where relevant, food composition tables from the UK Nutrient Databank (UKNDB), which includes food composition data most relevant to the time when UK Biobank participants completed the dietary assessments.

### 2.4. CALIPER UK workshop

The CALIPER UK Study team held a workshop in May 2021 with invited researchers from the WCRF, National Cancer Institute in the United States, Oxford University, Wageningen University, Radboud University and Health Research Institute of the Balearic Islands, including both those who contributed to the creation of the standardised scoring system as well as researchers applying this scoring system in cohorts worldwide. The aim of this workshop was to reach consensus on how to operationalise the scoring system using data available within UK Biobank.

## 3. Methods

### 3.1. Operationalisation of the standardised scoring system to assess adherence to the cancer prevention recommendations using UK Biobank data

A summary of the operationalisation of the standardised scoring system, including the scoring system cut-offs and the UK Biobank data used, can be found in Table 1. Operationalisation of each component of the scoring system is described in more detail below.

**Table 1:** Standardised scoring system used to assess adherence to the 2018 WCRF/AICR Cancer Prevention Recommendations, as devised by Shams-White et al. (2).

2018 WCRF/AICR Recommendation	Operationalization of Recommendations	Points	Original scoring system cut-offs
1. Be a healthy weight	**BMI (kg/m** ^**2**^ **)**		**BMI (kg/m** ^**2**^ **)**
	18.5–24.9	0.5	18.5–24.9
	25–29.9	0.25	25–29.9
	<18.5 or ≥ 30	0	<18.5 or ≥ 30
	**Waist circumference (cm (in))**		**Waist circumference (cm (in))**
	Men: <94 (<37) Women: <80 (<31.5)	0.5	Men: <94 (<37) Women: <80 (<31.5)
	Men: 94–<102 (37–<40) Women: 80–<88 (31.5–<35)	0.25	Men: 94–<102 (37–<40) Women: 80–<88 (31.5–<35)
	Men: ≥102 (≥40) Women: ≥88 (≥35)	0	Men: ≥102 (≥40) Women: ≥88 (≥35)
2. Be physically active	**Total moderate-vigorous physical activity (MET min/wk)**		**Total moderate-vigorous physical activity (min/wk)** ^**1**^
	≥600	1	≥150
	300–<600	0.5	75–<150
	<300	0	<75
3. Eat a diet rich in wholegrains, vegetables, fruit and beans	**Fruits and vegetables (g/day)**		**Fruits and vegetables (g/day)**
	≥400	0.5	≥400
	200–<400	0.25	200–<400
	<200	0	<200
	**Total fibre (g/day) (AOAC definition)**		**Total fibre (g/day) (AOAC definition)**
	≥30	0.5	≥30
	15–<30	0.25	15–<30
	<15	0	<15
4. Limit consumption of “fast foods” and other processed foods high in fat, starches or sugars	**Percent of total kcal from ultra-processed foods (aUPFs)**		**Percent of total kcal from ultra-processed foods (aUPFs)**
	Tertile 1 (lowest)	1	Tertile 1 (lowest)
	Tertile 2	0.5	Tertile 2
	Tertile 3 (highest)	0	Tertile 3 (highest)
5. Limit consumption of red and processed meat	**Total red meat and processed meat (g/wk)**		**Total red meat and processed meat (g/wk)**
	Red meat ≤500 and processed meat <21	1	Red meat ≤500 and processed meat <21
	Red meat ≤500 and processed meat 21–<100	0.5	Red meat ≤500 and processed meat 21–<100
	Red meat >500 or processed meat ≥100	0	Red meat >500 or processed meat ≥100
6. Limit consumption of sugar-sweetened drinks	**Total sugar-sweetened drinks (g/day):**		**Total sugar-sweetened drinks (g/day):**
	0	1	0
	>0–≤250	0.5	>0–≤250
	>250	0	>250
7. Limit alcohol consumption	**Total ethanol (UK guidelines) (units/week)**		**Total ethanol (US guidelines) (ethanol, g/day)**
	0	1	0
	≤14 units per week	0.5	>0–≤28 (2 drinks) males and ≤ 14 (1 drink) females
	> 14 units per week	0	>28 (2 drinks) males and > 14 (1 drink) females

1 Our cut-offs in MET min/wk are equivalent to those in the standardised scoring system in min/wk.

#### 3.1.1. Be a healthy weight

Anthropometric data on body mass index (BMI; data-field 21001) and waist circumference (data-field 48) were used to operationalise this recommendation. These measurements were collected at the assessment centre at the baseline study visit by trained staff using standard protocols. Weight was measured to the nearest 0.1 kg using the Tanita BC-418 MA body composition analyser and height using a Seca 202 height measure. BMI was calculated from weight and height data using the formula BMI = weight (kg)/height (m)^2^. Participants within the “normal” BMI range (18.5–24.9 kg/m^2^) were classed as fully meeting this sub-recommendation and given a score of 0.5 points. Participants with a BMI classed as “overweight”, who met the sub-recommendation partially, were given 0.25 points, and participants who were underweight (<18.5 kg/m^2^) or obese (≥30 kg/m^2^) were given 0 points.

Waist circumference was measured at the natural indent (or umbilicus if the natural indent could not be located) using a Seca 200 tape measure. The creators of the standardised scoring system derived the cut-points for the waist circumference sub-recommendation based on guidelines from the 2018 WCRF/AICR Cancer Prevention Recommendations, the Center for Disease Control and Prevention (14) and the U.S. National Heart, Lung, and Blood Institute (15). Male and female participants with waist circumferences <94 cm and < 80 cm, respectively, fully adhered to the waist circumference sub-recommendation and were given 0.5 points. Male participants with waist circumferences between 94 – 102 cm and female participants with waist circumferences between 80 and 88 cm were scored 0.25 points. Participants with waist circumferences ≥102 cm for males and ≥88 cm for females scored 0 points. The scores for the sub-recommendations on BMI and waist circumference were summed for a maximum score of 1 point for the “be a healthy weight” recommendation.

#### 3.1.2. Be physically active

The cut-offs for this recommendation are based on the WHO and U.S. Physical Activity Guidelines which advise adults to engage in at least 150 min of moderate-intensity aerobic physical activity or at least 75 min of vigorous-intensity physical activity per week (16). These guidelines are in line with those in the UK (17) and, therefore, relevant for a UK-based cohort.

Physical activity was self-reported and data were collected at the assessment centre study visit using a validated short form of the International Physical Activity Questionnaire (IPAQ) (18). The questionnaire asked participants about the frequency, intensity and duration of walking, moderate-intensity and vigorous-intensity physical activity during last month. Time spent in moderate to vigorous physical activity (MVPA), were reported in metabolic equivalents of task per week (MET-h/week). Briefly, the number of minutes per day reported for each level of activity was multiplied by the assigned MET equivalent (4 and 8 MET hours for moderate and vigorous physical activity, respectively) and converted to MET hours per week. Participants undertaking at least 600 MET/min per week (equivalent to 150 min of MVPA per week) were given 1 point, between 300 and 600 MET/min per week (equivalent to 75–150 min of MVPA per week) were given 0.5 points, and less than 300 MET/min per week (equivalent to less than 75 min of MVPA per week) were given 0 points. It should be noted that the cut-offs used in this study, where MVPA data are expressed in MET/min per week, are equivalent to those applied in the standardised scoring system (in min/wk).

#### 3.1.3. Eat a diet rich in wholegrains, vegetables, fruit, and beans

The wholegrains, vegetables, fruit and beans sub-score operationalises two goals pertaining to A. fruit and vegetable and B. fibre intake, described below.

##### 3.1.3.1. Eat a diet high in all types of plant foods including at least five portions or servings (at least 400 g or 15 oz in total) of a variety of non-starchy vegetables and fruit every day

Data on fruit and vegetable intake in the last 24 h (obtained using 24 h dietary assessment data) were used to assess adherence to this sub-recommendation. Information on the data-fields for the included fruits and vegetables can be found in the Supplementary methods. Due to the standardised scoring system’s focus on non-starchy vegetables within the fruits and vegetables sub-component (2), we excluded vegetables such as potatoes (fried, boiled/baked and mashed), sweet potatoes and butternut squash as well as beans and pulses. However, these foods were included when estimating dietary fibre intake for the fibre sub-component (please see below). Further, we did not include guacamole, found within the spreads and sauces category (data-field 20088). This is because the question simply asked whether or not items from a list of 19 spreads and sauces were consumed, so no information is available on the frequency of intake or portion size.

We used the frequency data and standard portion sizes for each food item (13) to calculate the mean intake in grams per day, and summed these to create a total intake of fruits and vegetables in grams per day. Where standard portion sizes were not defined for “Vegetable pieces” (data-field 104070), we allocated this portion as 60 g, which is the same as a standard portion of “Other vegetables” (data-field 104380). Participants who consumed at least 400 g of fruits and vegetables per day were given 0.5 points, those who consumed between 200 – 400 g were given 0.25 points, and those consuming less than 200 g per day scored 0 points.

##### 3.1.3.2. Consume a diet that provides at least 30 g/day of fibre from food sources

To operationalise the total dietary fibre intake component of the score, we used the 24 h dietary assessment nutrient data on Englyst fibre intake (data-field 100009). To estimate dietary fibre intake using the Association of Official Analytical Chemists (AOAC) method, we multiplied the dietary fibre variable, derived using the Englyst method, by a conversion factor of 1.33 as described by Lunn and Buttriss (19). Participants consuming ≥30 of dietary fibre per day were given 0.5 points, those consuming between 15 and 30 g per day were given 0.25 points and those consuming less than 15 g per day were given 0 points.

#### 3.1.4. Limit consumption of “fast foods” and other processed foods high in fat, starches or sugars

Shams-White and colleagues captured adherence to the recommendation on “fast” and processed foods using an adapted version of the NOVA classification system, which categorises foods according to the extent and purpose of processing (20). Group 1 of the NOVA classification includes foods that are unprocessed or minimally processed such as fruits, seeds, eggs and milk. Group 2 includes processed culinary ingredients, obtained directly from group 1 foods or from nature by processes such as pressing and milling, for example salt, sugar, vegetable oils and butter. Group 3 are processed foods, for example canned vegetables, salted nuts, smoked meats and cheeses, and unpackaged freshly-made breads. Group 4 are ultra-processed foods (UPFs) and drinks, which typically have five or more ingredients and undergo ultra-processing, for example to produce products that are ready to eat and have hyper-palatability. Examples of UPFs include carbonated (fizzy) drinks, confectionery (e.g., chocolate bars), breakfast cereals, ready meals such as pizzas and chicken nuggets, instant noodles, and mass-produced packaged breads and buns.

Firstly, we categorised the food variables available for the 24 h-dietary assessment data according to the NOVA classification system. An adapted UPF (aUPF) variable was created from the foods classified as Group 4 (ultra-processed), excluding food items already accounted for in other score components (i.e., sugar-sweetened drinks, processed meats and alcohol) to avoid double penalisation as described by Shams-White and colleagues (2, 21). Further information about the foods included, and the allocated portion sizes, can be found in the Supplementary Table 1. We acquired energy values (per 100 g) for these foods from the UKNDB, taking into account the food codes that best reflected the Oxford WebQ items as updated by Perez-Cornago et al., and the percentage allocation of each food code to each Oxford WebQ food item (13). We used these data to determine energy in kcals per standard portion size. Intake frequency data were multiplied by the energy value per standard portion size for each food item, and then summed to generate a variable for total energy intake from aUPFs. The energy intake variable (data-field 100002) was used to calculate the proportion of total daily energy intake from aUPFs.

Since there are no recommended cut-offs or guidelines for the consumption of UPFs, Shams-White and colleagues applied a subjective approach awarding points according to tertiles (2, 21). Participants in the highest tertile, consuming the highest amount of energy from aUPFs, scored 0 points, those in the middle tertile were given 0.5 point and those in the lowest tertile were given 1 point. The use of tertiles (and, hence, an approach which “ranks” individuals) to score this component overcomes discrepancies due to variation in i) aUPFs consumed in different countries and cultures, ii) how different dietary assessment methods affect estimates of aUPF consumption and iii) how aUPF consumption is expressed (for example as a proportion of total energy intake or in grams per day) (2).

#### 3.1.5. Limit consumption of red and processed meat

At our CALIPER UK workshop, we decided that for the red and processed meat recommendation, data expressed as frequency per week would be better than those obtained using the 24 h dietary assessment to capture usual intake, because red and processed meat may not be eaten on a daily basis. Therefore, data from the touchscreen FFQ-based questionnaire were used to operationalise the recommendation for red and processed meat intake.

The meat-related questions in the touchscreen questionnaire asked, “How often do you eat beef (data-field 1369)? (Do not count processed meats),” “How often do you eat lamb/mutton (data-field 1379)? (Do not count processed meats)” and “How often do you eat pork (data-field 1389)? (Do not count processed meats).” Participants were able to answer: “never,” “less than once a week,” “once a week,” “2–4 times a week,” “5–6 times a week,” “once or more daily,” “do not know” or “prefer not to answer.” As described by Bradbury et al. (10), the following intake frequencies were applied: “never” = 0, “less than once per week” = 0.5, “once per week” = 1, “2–4 times per week” = 3, “5–6 times per week” = 5.5, “once or more daily” = 7. Data coded as – 1 (corresponding to “do not know”) or – 3 (corresponding to “prefer not to answer”) were recoded as missing. The intakes of beef (data-field 1369), pork (data-field 1389) and lamb/mutton (data-field 1379) in grams per week were calculated by multiplying the frequency by a standard portion size of 120 g (13). A total red meat intake (g/wk) was calculated by adding each of these meat items together.

To assess processed meat intake, the answers to the question “How often do you eat processed meats (such as bacon, ham, sausages, meat pies, kebabs, burgers, chicken nuggets)?” (data-field 1349) were used. Intake frequencies were applied as described for red meat above. To assign a portion size for processed meats, we used the portion sizes detailed by Perez-Cornago et al. (13), where available (i.e., for bacon, ham, sausages, burgers and nuggets). For chicken nuggets, it was assumed that 56% of the portion was meat, as described by Stewart et al. (22). For pies, an average of the portion sizes of the pies included by Stewart et al. was used (43 g per portion). Because the touchscreen questionnaire asked about a range of processed foods that are typically consumed in different amounts in the UK, a weighted average was calculated using data on consumption of these foods from the National Diet and Nutrition Survey (NDNS) (23). This calculated weighted mean portion size (52.5 g) is similar to the unweighted mean (50.8 g). Details of the processed meat portion size calculations can be found in the Supplementary Table 2.

Participants were classed as fully adherent to this recommendation, and allocated 1 point, if their total red meat intake was 500 g or less per week and processed meat intake was less than 21 g per week. Participants who partially adhered to this recommendation, who consumed ≤500 g red meat per week but 21 g – <100 g of processed meat per week were given 0.5 points. Zero points were given to participants who did not adhere to the recommendation and consumed either >500 g red meat per week or ≥100 g processed meat per week.

#### 3.1.6. Limit consumption of sugar-sweetened drinks

Responses to the question “How much of the following did you drink yesterday?” and the intake of the following drinks were used to assess adherence to the recommendation on sugar-sweetened drinks: carbonated (fizzy) drinks (data-field 100170), fruit drinks, squash or cordial (data-field 100180), dairy/yoghurt-based smoothie (data-field 100230), flavoured milk (data-field 100530), hot chocolate (data-field 100550) and fruit smoothie (data-field 100220). Participants could answer the following: “none”, “1/2”, “1”, “2”, “3”, “4”, “5” or “6+”. Values for participants who answered “none” were coded as “0”, “1/2” were recoded to “0.5” and “6+” were recoded to “6”.

Intakes of these drinks were summed to create a mean sugar-sweetened drink intake per week. Assuming that a standard portion (one glass/carton/250 ml) equates to 250 g, participants who drank on average > 1 sugar-sweetened drink per day were allocated 0 points, those who consumed ≤1 scored 0.5 points, and those who did not consume sugar-sweetened drinks scored 1 point.

In line with other studies that have operationalised the scoring system (19), and following agreement on this approach during our CALIPER UK Workshop, we did not include sugar added to drinks by participants (such as sugar added to tea or coffee). This was decided to avoid unnecessary penalisation for the sugar-sweetened drinks recommendation as the Oxford WebQ does not allow for an accurate calculation of total sugar added to hot drinks. For example, a participant can select that they added a “varied” amount of sugar to teas, infusions and coffees throughout the day, if they drank more than one serving per day.

#### 3.1.7. Limit alcohol consumption

Since Shams-White and colleagues advise use of national guidelines or definitions regarding what constitutes an alcoholic drink (i.e., alcohol content and serving size) (21), we used UK national cut-offs to operationalise the alcohol recommendation (24).

The number of units of each alcoholic drink consumed per week were calculated from responses to the touchscreen questionnaire, i.e., for red wine (data-field 1568), white wine or champagne (data-field 1578), beer or cider (data-field 1588), spirits or liqueurs (data-field 1598), fortified wine (data-field 1608) and other alcoholic drinks such as alcopops (data-field 5364). The serving sizes corresponding to the question and units per serving, from the NHS website,^1^ are given in Supplementary Table 3. The number of units per week were calculated by multiplying the frequency of intake per week by the number of units corresponding to each drink. If a participant answered “Do not know” (coded as – 1) or “Prefer not to answer” (coded as – 3), they were coded as missing. The total number of units of alcohol consumed per week were calculated by summing the number of units consumed per week of red wine, white wine or champagne, beer or cider, spirits or liqueurs fortified wine and other alcoholic drinks such as alcopops.

Participants who consumed more than 14 units of alcohol per week were given 0 points, those who consumed >0 – ≤14 units of alcohol per week were given 0.5 points and those adhering fully to the recommendation were given 1 point. Further, participants who answered “never” (coded as “6”) or “special occasions only” (coded as “5”) to the question “About how often do you drink alcohol?” (data-field 1558) were allocated 1 point. Participants who answered “one to three times a month” (coded as “4”) to this question were allocated 0.5 points. This is in line with the further guidance on operationalisation of the standardised scoring system by Shams-White and colleagues, which recommends that, given the limited evidence comparing non-drinkers to very rare drinkers, participants who consume up to one drink per month should be classed as non-drinkers, and those consuming more than one drink per month should fall within the 0.5 and 0 point categories, depending on the amount of alcohol consumed (21).

For future sensitivity analyses, we have also calculated a score using the cut-offs described in the standardised scoring system, based on US guidelines (28 g of ethanol (2 drinks) and 14 g of ethanol (1 drink) per day for males and females, respectively) (2).

#### 3.1.8. Total score calculation

A total score was calculated by summing the points for each of the seven recommendations, with a range of 0–7 points. We were not able to assess adherence to the eighth optional recommendation for mothers to breastfeed their baby, if they can, as these data were not collected by the UK Biobank. A separate 5-point scoring system based on the touchscreen questionnaire was also calculated, and details of this calculation are described in the Supplementary methods.

## 4. Anticipated results

**Figure 1:**
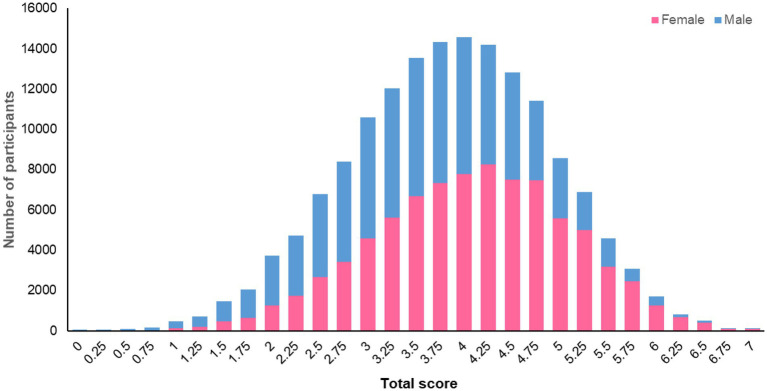
Distribution of total adherence scores for male and female UK Biobank participants.

The methodology (described above) for fully operationalising the standardised scoring system (2) for assessing adherence to the 2018 WCRF/AICR Cancer Prevention Recommendations allows for the calculation of a “total adherence score” for participants in the UK Biobank who completed at least one 24 h dietary assessment and for whom we had data at baseline for BMI, waist circumference, physical activity and diet from the touchscreen questionnaire (*n* = 158,415). The mean total score for these 158,415 participants was 3.9 (SD 1.0) points and ranged from 0 to 7 points. The distribution of total scores for female and male participants is illustrated in Figure 1. This total score will be used to investigate relationships between adherence to the WCRF/AICR Cancer Prevention Recommendations and the risk of, and survival from, cancers, as well as other non-communicable diseases. The CALIPER UK Study will explore potential refinements to the score, such as changing the data type or cut-offs used to assess adherence to a recommendation and the weighting given to each score component in calculating the total score.

In addition, we have devised a 5-point, FFQ-based score using the baseline touchscreen questionnaire data, which allows assessment of adherence to five of the recommendations concerning (i) body weight, (ii) physical activity, (iii) fruits, vegetables and fibre intake, (iv) red and processed meats intake, and (v) alcohol consumption, in a larger subset of UK Biobank participants (*n* = 314,616). The mean FFQ-based score based on this 5-point system is 2.64 (SD 0.91) and there was a strong correlation between the full “total score” and the 5-point score (Spearman’s rho = 0.796, *p* < 0.0001, *n* = 127,667). Using the modified 5-point score that is available for a larger subset of participants (*n* = 314,616) will provide greater statistical power for investigations of associations between the adherence score and health-related outcomes.

## 5. Discussion

We have described the methodology applied, and data used, to operationalise a standardised scoring system for assessing adherence to the 2018 WCRF/AICR Cancer Prevention Recommendations for participants in the UK Biobank prospective cohort study, with the aim of promoting transparency and enhancing reproducibility of findings. Our methodology included running a CALIPER UK Workshop with experts from across the world to allow us to identify how best to operationalise the standardised scoring system with the data available and challenges faced within the UK Biobank. These experts included creators of the standardised scoring system, researchers with substantial experience in processing and using UK Biobank dietary data, members of the WCRF who contributed to the development of the Cancer Prevention Recommendations and researchers who are operationalising the standardised scoring system in other cohorts worldwide. Discussions and decisions made at this Workshop included defining the food variables to be classed as aUPFs using the NOVA classification system and the use of alternative cut-offs based on national guidelines to assess adherence to the recommendation on alcohol consumption.

Using UK Biobank data, we operationalised all components of the score. This is in contrast with some other studies that had missing information on, for example, some of the anthropometric measurements (5, 9) or physical activity (25). As advised by Shams-White and colleagues (2), we applied country-specific guidelines and cut-offs where applicable, for example for the alcohol recommendation where, in the UK, one unit of alcohol contains 8 g of ethanol and both men and women are advised not to drink more than 14 units per week.^2^ In future studies, we will explore differences in the total adherence score, including the strengths of associations with cancer incidence, when using other cut-offs including those described by Shams-White et al. (2). In addition, we have created a modified, 5-point touchscreen questionnaire-based score that will allow us to i) compare adherence scores derived from different methods of assessment of nutritional intake and ii) run investigations of associations between adherence score and health outcomes in a larger sample of UK Biobank participants (*n* = 314,616) who do not all have 24 h dietary assessment data. We found a strong and statistically significant correlation between the full “total score” and the 5-point score in 127,667 UK Biobank participants. As already described, some previous studies have also reported calculating partial or modified scores (9, 26).

A strength of this study is the alignment with other analyses of UK Biobank that have used standard portion sizes to estimate intakes of energy and of Englyst fibre from the 24 h dietary assessment data (13). Where standard portion sizes were unavailable, for example for the processed meat food items, we used data from the NDNS to estimate portion sizes. We have applied a conservative approach and minimised use of assumptions throughout. For example, because of the lack of information on intakes of specific foods, e.g., guacamole, we did not include food items from the spreads and sauces category (data-field 20088) in the “fruits and vegetables” sub-recommendation, nor did we include brown sauce and cheese sauce in the aUPF recommendation. However, inclusion of even one serving of a standard portion size of 26 g of guacamole per day is unlikely to make a substantial difference to participant scores for this sub-recommendation.

As advised by Shams-White and colleagues (2), we have considered the utility of the dietary data obtained from the two assessment methods (touchscreen questionnaire versus 24 h dietary assessment) in the UK Biobank to operationalise each score component. As a consequence, we have used a combination of the two assessment methods, with the data collected at different time points and also over time for multiple 24 h dietary assessment, which is a limitation of our study. For some foods not consumed daily, such as red and processed meats, we used data from the touchscreen questionnaire, whereas for some items such as sugar-sweetened beverages, we used the 24 h dietary assessment data because information on intake of these beverages was not collected in the touchscreen questionnaire. Nonetheless, Bradbury and colleagues have observed good agreement between the dietary data collected using the two approaches and have shown that the touchscreen questionnaire method reliably ranks participants according to the intake of main foods and food groups (10). Furthermore, there was good reproducibility between estimates of habitual diet estimated using responses to the touchscreen questionnaire at baseline and those completed 4 years later at the repeat assessment centre visit, suggesting no major long-term changes in diet during this period (10). However, participants who completed the repeat touchscreen questionnaire or at least one of the follow-up 24 h dietary assessments were more likely to be more educated and less likely to smoke compared with the full UK Biobank cohort (10).

In our future analyses we will consider adjusting for such sociodemographic factors; however, this is more of a concern for external generalisability rather than for internal validity of our findings. Although completion of two 24 h dietary assessments may not be sufficient to capture habitual intakes precisely, including participants with data from at least two 24 h dietary assessments is a reasonable compromise to avoid losing too many participants in future studies of associations with cancer and other health outcomes. When compared with the general population, participants in UK Biobank were less likely to be obese, drank less alcohol and were less likely to be smokers (27), thus our findings may not be generalisable to all adults in the UK.

Lastly, this analysis utilised self-reported data for some score components, including the dietary and physical activity data, which may be prone to recall bias or misreporting. However, a strength of this study is that the anthropometric measurements made in the UK Biobank and used to assess adherence to the recommendation to maintain a healthy body weight were collected by trained staff using standardised procedures at the assessment centre visit.

In conclusion, we have used robust methodology to apply the standardised scoring system created by Shams-White and colleagues (2) to assess adherence to the WCRF/AICR Cancer Prevention Recommendations, within the UK Biobank. Here, we are the first to describe in detail how we have operationalised the adherence scoring system in order to allow for transparency and reproducibility and aid interpretation of our future findings. Since UK Biobank is an internationally significant cohort study that is being used extensively to investigate links between lifestyle behaviours and health-related outcomes, such as cancer, we hope that this will be useful for other researchers using UK Biobank data, as well as to provide guidance on operationalising the scoring system in other studies. Our future work will investigate relationships between adherence score and cancer risk and survival within this UK cohort. In addition, as encouraged by Shams-White and colleagues (2, 21), we will explore whether assigning different weightings to each recommendation within the scoring system affects its utility. We will also investigate the impact of changes in how each component is assessed, for example using alternative measures of adiposity to assess adherence to the “be a healthy body weight” recommendation (28), on the scoring system.

## Data availability statement

The datasets presented in this article are not readily available because data are available upon request from UK Biobank (www.ukbiobank.ac.uk). Requests to access the datasets should be directed to john.mathers@newcastle.ac.uk.

## Ethics statement

The studies involving human participants were reviewed and approved by The UK Biobank was conducted in accordance with the Declaration of Helsinki and was approved by the North West Multi-Centre Research Ethics Committee (REC reference: 12/NW/03820). The patients/participants provided their written informed consent to participate in this study.

## Author contributions

FM, SP-S, CC-M, LS, and JM contributed to the conception and design of the study. FM, SP-S, LL, FH, AP-C, MS-W, MZ, EK, RW, GM, MW, DR, CC-M, LS, and JM attended and actively contributed to the CALIPER UK Workshop, which aided the design and execution of the study. FM wrote the first draft of the manuscript. JM, SP-S, LS, FH, CC-M, AP-C, MS-W, MZ, EK, RW, DR, and GM revised the manuscript. All authors contributed to the article and approved the submitted version.

## Funding

This research was funded by grant number IIG_FULL_2020_032 from the Wereld Kanker Onderzoek Fonds (WKOF), as part of the World Cancer Research Fund International grant programme. SPS received financial support from the Chilean Government for their PhD (ANID-Becas Chile, project 72200012).

## Conflict of interest

The authors declare that the research was conducted in the absence of any commercial or financial relationships that could be construed as a potential conflict of interest.

## Publisher’s note

All claims expressed in this article are solely those of the authors and do not necessarily represent those of their affiliated organizations, or those of the publisher, the editors and the reviewers. Any product that may be evaluated in this article, or claim that may be made by its manufacturer, is not guaranteed or endorsed by the publisher.

## Footnotes

^1^ https://www.nhs.uk/live-well/alcohol-advice/calculating-alcohol-units/

^2^ https://www.nhs.uk/live-well/alcohol-support/calculating-alcohol-units/

## Abbreviations

AICR: American Institute for Cancer Research
AOAC: Association of Official Analytical Chemists
aUPF: adapted ultra-processed foods
BMI: body mass index
IPAQ: International Physical Activity Questionnaire
MET: metabolic-equivalent
MVPA: Moderate to vigorous physical activity
NDNS: National Diet and Nutrition Survey
PA: physical activity
UPF: ultra-processed food
WCRF: World Cancer Research Fund

## References

[1] Research WCRFAIfC. 2018. Diet, nutrition, physical activity and cancer: a global perspective. Continuous update project expert report. Available from: http://dietandcancerreport.org (Accessed December 9, 2021).

[2] Shams-White MM Brockton NT Mitrou P Romaguera D Brown S Bender A . Operationalizing the 2018 World Cancer Research Fund/American Institute for Cancer Research (WCRF/AICR) cancer prevention recommendations: a standardized scoring system. Nutrients. (2019) 11:1572. doi: 10.3390/nu11071572, PMID: 31336836 PMC6682977

[3] van Zutphen M Boshuizen HC Kenkhuis MF Wesselink E Geijsen A de Wilt JHW . Lifestyle after colorectal cancer diagnosis in relation to recurrence and all-cause mortality. Am J Clin Nutr. (2021) 113:1447–57. doi: 10.1093/ajcn/nqaa394, PMID: 33677488 PMC8168353

[4] Tollosa DN Holliday E Hure A Tavener M James EL. Multiple health behaviors before and after a cancer diagnosis among women: a repeated cross-sectional analysis over 15 years. Cancer Med. (2020) 9:3224–33. doi: 10.1002/cam4.2924, PMID: 32134568 PMC7196049

[5] Zhang ZQ Li QJ Hao FB Wu YQ Liu S Zhong GC. Adherence to the 2018 World Cancer Research Fund/American Institute for Cancer Research cancer prevention recommendations and pancreatic cancer incidence and mortality: a prospective cohort study. Cancer Med. (2020) 9:6843–53. doi: 10.1002/cam4.3348, PMID: 32716590 PMC7520356

[6] Shams-White MM Brockton NT Mitrou P Kahle LL Reedy J. The 2018 World Cancer Research Fund/American Institute for Cancer Research (WCRF/AICR) score and all-cause, cancer, and cardiovascular disease mortality risk: a longitudinal analysis in the NIH-AARP diet and health study. Curr Dev Nutr. (2022) 6:6006009. doi: 10.1093/cdn/nzac096 PMC9217081 35755938

[7] Olmedo-Requena R Lozano-Lorca M Salcedo-Bellido I Jimenez-Pacheco A Vazquez-Alonso F Garcia-Caballos M . Compliance with the 2018 World Cancer Research Fund/American Institute for Cancer Research cancer prevention recommendations and prostate cancer. Nutrients. (2020) 12:768. doi: 10.3390/nu12030768 32183345 PMC7146507

[8] Barrios-Rodriguez R Toledo E Martinez-Gonzalez MA Aguilera-Buenosvinos I Romanos-Nanclares A Jimenez-Moleon JJ. Adherence to the 2018 World Cancer Research Fund/American Institute for Cancer Research recommendations and breast cancer in the SUN project. Nutrients. (2020) 12:2076. doi: 10.3390/nu12072076, PMID: 32668662 PMC7400833

[9] Turati F Dalmartello M Bravi F Serraino D Augustin L Giacosa A . Adherence to the World Cancer Research Fund/American Institute for Cancer Research recommendations and the risk of breast cancer. Nutrients. (2020) 12:607. doi: 10.3390/nu12030607, PMID: 32110887 PMC7146587

[10] Bradbury KE Young HJ Guo W Key TJ. Dietary assessment in UK biobank: an evaluation of the performance of the touchscreen dietary questionnaire. J Nutr Sci. (2018) 7:e6. doi: 10.1017/jns.2017.66, PMID: 29430297 PMC5799609

[11] UKBiobank. 2007 UK biobank. Protocol for a large-scale prospective epidemiological resource. Available from: http://www.ukbiobank.ac.uk/wp-content/uploads/2011/11/UK-Biobank-Protocol.pdf

[12] Liu B Young H Crowe FL Benson VS Spencer EA Key TJ . Development and evaluation of the Oxford WebQ, a low-cost, web-based method for assessment of previous 24 h dietary intakes in large-scale prospective studies. Public Health Nutr. (2011) 14:1998–2005. doi: 10.1017/S1368980011000942, PMID: 21729481

[13] Perez-Cornago A Pollard Z Young H van Uden M Andrews C Piernas C . Description of the updated nutrition calculation of the Oxford WebQ questionnaire and comparison with the previous version among 207,144 participants in UK biobank. Eur J Nutr. (2021) 60:4019–30. doi: 10.1007/s00394-021-02558-4, PMID: 33956230 PMC8437868

[14] CDC (2022). Center for Disease Control and Prevention (CDC) healthy weight: assessing your weight. Available from: https://www.cdc.gov/healthyweight/assessing/index.html (Accessed July 7, 2022).

[15] NIH (2022). National Heart, Lung, and Blood Institute: assessing your weight and health risk. Available from: https://www.nhlbi.nih.gov/health/educational/lose_wt/risk.htm (Accessed July 7, 2022).

[16] U.S. Department of Health and Human Services. Physical activity guidelines for Americans. 2nd Edn. Washington, DC: U.S. Department of Health and Human Services. (2018).

[17] Department of Health Alcohol Guidelines Review 2019. UK Chief Medical Officers’ Physical Activity Guidelines. Available from: https://www.gov.uk/government/publications/physical-activity-guidelines-uk-chief-medical-officers-report

[18] Craig CL Marshall AL Sjostrom M Bauman AE Booth ML Ainsworth BE . International physical activity questionnaire: 12-country reliability and validity. Med Sci Sports Exerc. (2003) 35:1381–95. doi: 10.1249/01.MSS.0000078924.61453.FB, PMID: 12900694

[19] Lunn J Buttriss JL. Carbohydrates and dietary fibre. Nutr Bull. (2007) 32:21–64. doi: 10.1111/j.1467-3010.2007.00616.x

[20] Monteiro CA Cannon G Levy R Moubarac JC Jaime P Martins AP . NOVA. The star shines bright. World Nutrition. (2016) 7:28–38.

[21] Shams-White MM Romaguera D Mitrou P Reedy J Bender A Brockton NT. Further guidance in implementing the standardized 2018 World Cancer Research Fund/American Institute for Cancer Research (WCRF/AICR) score. Cancer Epidemiol Biomark Prev. (2020) 29:889–94. doi: 10.1158/1055-9965.EPI-19-1444, PMID: 32152215

[22] Stewart C Frie K Piernas C Jebb SA. Development and reliability of the Oxford meat frequency questionnaire. Nutrients. (2021) 13:922. doi: 10.3390/nu13030922, PMID: 33809192 PMC7999625

[23] NDNS. National Diet and Nutrition Survey. Headline results from years 1,2,3 and 4 (combined) of the rolling Programme (2008/2009–2011/12). Table 5.2. 2016. Available at: https://www.gov.uk/government/publications/physical-activity-guidelines-uk-chief-medical-officers-report

[24] Alcohol Guidelines Review – Report from the Guidelines Development Group to the UK Chief Medical Officers 2016. Report from the guidelines development group to the UK chief medical officers.

[25] Geijsen A Kok DE van Zutphen M Keski-Rahkonen P Achaintre D Gicquiau A . Diet quality indices and dietary patterns are associated with plasma metabolites in colorectal cancer patients. Eur J Nutr. (2021) 60:3171–84. doi: 10.1007/s00394-021-02488-1, PMID: 33544207 PMC8354955

[26] Solans M Romaguera D Gracia-Lavedan E Molinuevo A Benavente Y Saez M . Adherence to the 2018 WCRF/AICR cancer prevention guidelines and chronic lymphocytic leukemia in the MCC-Spain study. Cancer Epidemiol. (2020) 64:101629. doi: 10.1016/j.canep.2019.101629, PMID: 31756676

[27] Fry A Littlejohns TJ Sudlow C Doherty N Adamska L Sprosen T . Comparison of sociodemographic and health-related characteristics of UK biobank participants with those of the general population. Am J Epidemiol. (2017) 186:1026–34. doi: 10.1093/aje/kwx246, PMID: 28641372 PMC5860371

[28] Parra-Soto S Malcomson FC Ho FK Pell JP Sharp L Mathers JC . Associations of a body shape index (ABSI) with cancer incidence, all-cause, and at 23 sites-findings from the UK biobank prospective cohort study. Cancer Epidemiol Biomark Prev. (2022) 31:315–24. doi: 10.1158/1055-9965.EPI-21-0591, PMID: 34853021

