# Supplementary material for: Operationalisation of a standardised scoring system to assess adherence to the World Cancer Research Fund/American Institute for Cancer Research cancer prevention recommendations in the UK biobank
Source: Front Nutr. 2023 Feb 10;10:1011786. doi: 10.3389/fnut.2023.1011786 (PMC9950547; doi:10.3389/fnut.2023.1011786)
Supplement: Supplementary file 1 [file Data_Sheet_1.docx]

**SUPPLEMENTARY MATERIALS**

**Supplementary Table 1: 24-hour dietary assessment food items included in the aUPF variable (NOVA group 4)**

| **Food name** | **UKBB data-field** | **Energy per portion (kcals)** | **Portion size (g)^a^** | **mean energy (kcals/100g)** |
| --- | --- | --- | --- | --- |
| Low calorie drink intake | 100160 | 2 | 330 | 1 |
| Low calorie hot chocolate intake | 100540 | 50 | 260 | 19 |
| Muesli intake | 100800 | 283 | 80 | 354 |
| Oat crunch intake | 100810 | 246 | 60 | 410 |
| Sweetened cereal intake | 100820 | 147 | 38 | 387 |
| Plain cereal intake | 100830 | 115 | 30 | 384 |
| Bran cereal intake | 100840 | 154 | 50 | 308 |
| Whole-wheat cereal intake | 100850 | 155 | 44 | 352 |
| Other cereal intake | 100860 | 169 | 44 | 384 |
| Sliced bread intake | 100950 | 103 | 36 | 287 |
| Bap intake | 101090 | 249 | 90 | 277 |
| Bread roll intake | 101160 | 169 | 60 | 282 |
| Naan bread intake | 101230 | 429 | 160 | 268 |
| Garlic bread intake | 101240 | 70 | 20 | 348 |
| Crispbread intake | 101250 | 38 | 10 | 381 |
| Oatcakes intake | 101260 | 59 | 13 | 453 |
| Other bread intake | 101270 | 122 | 45 | 271 |
| Double crust pastry intake | 101970 | 312 | 60 | 520 |
| Single crust pastry intake | 101980 | 160 | 30 | 534 |
| Crumble intake | 101990 | 336 | 70 | 480 |
| Pizza intake | 102000 | 408 | 150 | 272 |
| Pancake intake | 102010 | 206 | 110 | 188 |
| Scotch pancake intake | 102020 | 77 | 41 | 188 |
| Yorkshire pudding intake | 102030 | 60 | 25 | 239 |
| Indian snacks intake | 102040 | 111 | 40 | 278 |
| Croissant intake | 102050 | 242 | 60 | 404 |
| Danish pastry intake | 102060 | 491 | 110 | 446 |
| Scone intake | 102070 | 168 | 48 | 351 |
| Ice-cream intake | 102120 | 263 | 120 | 219 |
| Milk-based pudding intake | 102140 | 184 | 200 | 92 |
| Other milk-based pudding intake | 102150 | 158 | 60 | 264 |
| Soya dessert intake | 102170 | 151 | 125 | 121 |
| Fruitcake intake | 102180 | 475 | 140 | 339 |
| Cake intake | 102190 | 247 | 60 | 411 |
| Doughnut intake | 102200 | 224 | 60 | 374 |
| Sponge pudding intake | 102210 | 365 | 120 | 304 |
| Cheesecake intake | 102220 | 55 | 15 | 368 |
| Other dessert intake | 102230 | 96 | 60 | 159 |
| Chocolate bar intake | 102260 | 223 | 50 | 446 |
| White chocolate intake | 102270 | 261 | 50 | 521 |
| Milk chocolate intake | 102280 | 260 | 50 | 519 |
| Dark chocolate intake | 102290 | 255 | 50 | 510 |
| Chocolate-covered raisin intake | 102300 | 99 | 25 | 397 |
| Chocolate sweet intake | 102310 | 161 | 36 | 447 |
| Diet sweets intake | 102320 | 44 | 18 | 243 |
| Sweets intake | 102330 | 136 | 36 | 378 |
| Chocolate-covered biscuits intake | 102340 | 121 | 24 | 504 |
| Chocolate biscuits intake | 102350 | 80 | 17 | 472 |
| Sweet biscuits intake | 102360 | 81 | 17 | 474 |
| Cereal bar intake | 102370 | 181 | 44 | 411 |
| Other sweets intake | 102380 | 192 | 40 | 481 |
| Crisp intake | 102460 | 199 | 40 | 498 |
| Savoury biscuits intake | 102470 | 144 | 40 | 359 |
| Cheesy biscuits intake | 102480 | 198 | 40 | 494 |
| Powdered/instant soup intake | 102530 | 74 | 200 | 37 |
| Canned soup intake | 102540 | 132 | 220 | 60 |
| Snackpot intake | 102760 | 53 | 40 | 132 |
| Low fat cheese spread intake | 102850 | 23 | 15 | 153 |
| Cheese spread intake | 102860 | 38 | 15 | 253 |
| Scotch egg intake | 102970 | 289 | 120 | 241 |
| Crumbed or deep-fried poultry intake | 103050 | 225 | 100 | 225 |
| Breaded fish intake | 103170 | 202 | 100 | 202 |
| Battered fish intake | 103180 | 354 | 150 | 236 |
| Vegetarian sausages/burgers intake | 103260 | 44 | 20 | 221 |
| Tofu/tempeh/TVP/soya mince intake | 103270 | 17 | 20 | 85 |
| Quorn intake | 103280 | 30 | 20 | 151 |
| Baked bean intake | 104000 | 107 | 135 | 80 |
| Fried potatoes intake | 104020 | 342 | 180 | 190 |
| Coleslaw intake | 104080 | 262 | 120 | 219 |

**Supplementary Table 2: Calculation of portion sizes assigned to processed meat food items reported in the touchscreen FFQ**

| **Food item** | **Allocated weighting^1^** | **Average portion size (g)** | **weighting*portion size** |
| --- | --- | --- | --- |
| Bacon and ham^2^ | 12.30% | 34.5 | 4.24 |
| Sausages | 21.20% | 30 | 6.36 |
| Pies | 23.20% | 43^3^ | 9.98 |
| Burgers + kebabs^4^ | 22.20% | 90.5 | 20.09 |
| Nuggets^5^ | 21.10% | 56^6^ | 11.82 |
|  | **Weighted mean portion** | **52.5g** |  |
|  | **Unweighted mean portion** | **50.8g** |  |

The Food Composition table standard portion sizes (13) were used for all food items with the exception of pies, where the portion sizes given by Stewart et al (2021)(22) (Supplementary file S2) were used and for kebabs where the FSA data(29) for ‘average portion, meat on skewer’ were used.

^1^ NDNS(23) data on the total quantities of each food item consumed (grams) per day: all consumers, for individuals aged 19-64 (2008/9 – 2011/12), were used to calculate the weighting allocated to each food item

^2^ Intake data are grouped together in the NDNS, thus the mean of the portion size of the two food items (bacon 46g and ham 23g) was used.

^3^ The mean meat (g) content was based on chicken pie (32g), lamb pie (44g), beef pie (40g), pork pie (33g), and game pie (63g)- information from Stewart et al. (2021)

^4^ Intake data are grouped together on the NDNS, thus, after disaggregating the meat content (burgers 91g and kebabs 90g), the mean of the portion sizes of two food items was used.

^5^ Used NDNS data for ‘coated chicken’

^6^ Derived using the proportion of meat content information from Stewart et al. (2021) (56%) and the FCT standard portion size for nuggets (100g)(30)

**Supplementary Table 3**: **Average weekly intake of alcoholic drinks from the touchscreen questionnaire and corresponding units per serving**

| **Alcoholic drink** | **UKBB data-field** | Serving size | **Units per serving (REF NHS website)** |
| --- | --- | --- | --- |
| Red wine | 1568 | 1 small glass (125ml) | 1.5 |
| White wine/champagne | 1578 | 1 small glass (125ml) | 1.5 |
| Beer/cider | 1588 | 1 pint | 2 units^a^ |
| Spirits/liqueurs | 1598 | Single small shot of spirits (25ml, ABV 40%) | 1 unit |
| Other alcoholic drinks e.g. Alcopops | 5364 | Alcopop (275ml, ABV 5.5%) | 1.5 |
| Fortified wine e.g. Sherry, port, vermouth | 1608 |  | 1 |

^a^ Based on a pint of lower-strength lager/beer/cider (ABV 3.6%)

**Supplementary Methods**

**3. “Eat a diet rich in wholegrains, vegetables, fruit, and beans”**

*3A “Eat a diet high in all types of plant foods including at least five portions or servings (at least 400 g or 15 oz in total) of a variety of non-starchy vegetables and fruit every day”*

Data on fruit and vegetable intake in the last 24 hours (obtained using 24-hour dietary assessment data) were used to assess adherence to this sub-recommendation. The following fruits and vegetables were included: mixed vegetables (data-field 104060), vegetable pieces (data-field 104070), coleslaw (data-field 104080), side salad (data-field 104090), avocado (data-field 104100), green beans (data-field 104120), broccoli (data-field 104140), cabbage/kale (data-field 104160), carrot (data-field 104170), cauliflower (data-field 104180), celery (data-field 104190), courgette (data-field 104200), cucumber (data-field 104210), garlic (data-field 104220), leek (data-field 104230), lettuce (data-field 104240), mushroom (data-field 104250), onion (data-field 104260), sweet pepper (data-field 104290), spinach (data-field 104300), sprout (data-field 104310), fresh tomato (data-field 104340), tinned tomatoes (data-field 104350), watercress (data-field 104370), other vegetables (data-field 104380), apple (data-field 104450), stewed fruit (data-field 104410), prunes (data-field 104420), dried fruit (data-field 104430), mixed fruit (data-field 104440), banana (data-field 104460), berry (data-field 104470), cherry (data-field 104480), grapefruit (data-field 104490), grapes (data-field 104500), mango (data-field 104510), melon (data-field 104520), orange (data-field 104530), satsuma (data-field 104540), peaches/nectarine (data-field 104550), pear (data-field 104560), pineapple (data-field 104570), plum (data-field 104580) and other fruits (data-field 104590).

**Calculation of touchscreen questionnaire-based 5-point score**

A modified, 5-point touchscreen questionnaire-based score was created to assess adherence to the following recommendations: ‘1. Be a healthy weight’, ‘2. Be physically active’, ‘3. Eat a diet rich in wholegrains, vegetables, fruit and beans’, 4. Limit consumption of red and processed meat and 5. Limit alcohol consumption’. Assessment of adherence to the recommendations ‘Be a healthy weight’, ‘Be physically active’ and ‘Limit consumption of red and processed meat’ and ‘Limit alcohol consumption’ were as already described for operationalisation of the standardised scoring system, which also included data from the touchscreen questionnaire. Therefore, operationalisation of the component ‘Eat a diet rich in wholegrains, vegetables, fruit and beans’, which includes two sub-recommendations on i) fruit and vegetable intake and ii) dietary fibre intake was added as described below.

*“Eat a diet high in all types of plant foods including at least five portions or servings (at least 400 g or 15 oz in total) of a variety of non-starchy vegetables and fruit every day”*

To estimate fruit and vegetable intake, we used data from the touchscreen questions about the daily consumption of fresh fruit (data-field 1309), dried fruit (data-field 1319), cooked vegetables (data-field 1289) and raw vegetables (data-field 1299). For example, the question on fresh fruit (data-field 1309) asked "About how many pieces of fresh fruit would you eat per day? (Count one apple, one banana, 10 grapes etc as one piece; put '0' if you do not eat any)". Data for participants who answered “Less than one” (coded as -10) were recoded as 0.5 servings. If a participant answered “Do not know” (coded as -1) or “Prefer not to answer” (coded as -3), they were coded as missing. Daily consumption was converted to intake in grams per day by multiplying the frequency by the corresponding mean portion (in grams), as shown in Supplementary Table 4. For fresh fruit (data-field 1309) and dried fruit (data-field 1319), we took the mean of the portion in grams of the food items e.g. apple, banana listed as examples on the question, using updated portion sizes from Perez-Cornago et al.(13) i.e. to yield a mean portion of 126g. For cooked (data-field 1289) and raw (data-field 1299) vegetables, as the question asked about heaped tablespoons rather than portions, data for the equivalent weight in gram was used as described by Bradbury and colleagues(10).

The total intake of fruits and vegetables in grams per day was calculated by summing the derived values for fresh fruit, dried fruit, cooked vegetables and raw vegetables. The aforementioned cut-offs were applied to allocated scores for the ‘fruits and vegetables’ sub-component of the scoring system.

**Supplementary Table 4:**

| **UKBB FFQ variable** |  | **Portion size (g)(13)** |
| --- | --- | --- |
| **Fresh fruit n_1309** | Apple | 112 |
|  | 1 banana | 100 |
|  | Grapes | 100 |
|  | Pear | 160 |
|  | Orange | 120 |
|  | **Mean** | **118** |
| **Dried fruit n_1319** | 1 prune | 8 |
|  | 1 apricot | 8 |
|  | 10 raisins | 15 |
|  | **Mean** | **10** |
| **Cooked vegetables n_1289** | **1 heaped tbsp** | **McCance & Widdowson weight of specified portion (g)** (10) |
|  | baked beans | 40 |
|  | peas | 30 |
|  | carrots | 40 |
|  | sweetcorn | 30 |
|  | **Mean** | **35** |
| **Raw vegetables n_1299** | **1 heaped tbsp** |  |
|  | baked beans | 40 |
|  | peas | 30 |
|  | carrots | 40 |
|  | sweetcorn | 30 |
|  | **Mean** | **35** |

*“Consume a diet that provides at least 30 g/day of fibre from food sources”*

To estimate dietary fibre intake from the touchscreen questionnaire data, a partial fibre score was calculated based on the intake of fresh fruit, dried fruit, raw vegetables, cooked vegetables, bread (taking into account bread type) and breakfast cereals (taking into account cereal type) as described by Bradbury and colleagues(31). The weight for raw vegetables was assumed to be the same as cooked vegetables.

If a participant responded ‘do not know’ or ‘prefer not answer’ for the bread type (data-field 1448) question, but reported their bread intake (data-field 1438), the average bread fibre content was assigned. The same was applied for breakfast cereal type (data-field 1468) question and corresponding breakfast cereal intake (data-field 1458). If a participant selected ‘do not know’ or ‘prefer not to answer’ for the questions on fresh fruit, dried fruit, raw vegetables or cooked vegetables for any of the remaining food items, they were coded as missing for the partial fibre score calculation. The fibre content per portion of the food (an approximate non-starch polysaccharide content(32)) was multiplied by the reported frequency of consumption (Supplementary Table 5). Touchscreen questionnaire responses that were ‘less than one’ were recoded to ‘0.5’. Participants who answered ‘0’ or ‘Less than one’ for slices of bread and bowls of breakfast cereal were not asked which type of bread or cereal they usually consumed so they were given 0g of fibre from these questions

For foods for which the touchscreen questionnaire asked about weekly consumption (bread and breakfast cereal), the frequency per week was converted to a daily average. Partial dietary fibre intake was estimated by adding the estimated fibre intake from fresh fruit, dried fruit, raw vegetables, cooked vegetables, bread and breakfast cereals.

As the partial fibre score(10) does not fully estimate total fibre intake (mean partial fibre score = 14.9g/d, n=385,356), cut-offs based on tertiles were applied for the fibre sub-component of the scoring system, as recommended by Shams-White and colleagues(21). Participants with the lowest partial fibre score (mean intake 9.0g/d) were given 0 points, those in the middle tertile (mean intake 14.4g) were given 0.25 points, and those in the highest tertile (mean intake 21.5g/d) scored 0.5 points.

**Supplementary Table 5.** Fibre content of the foods included in the dietary touchscreen questions used to estimate a partial dietary fibre score in UK Biobank as described by Bradbury and colleagues (10)

| **Food item** | **Portion specified in touchscreen question** | **Portion size(29)** | **Estimated non-starch polysaccharide content g/100g(32)** | **Estimated fibre content/portion (g)** |  |
| --- | --- | --- | --- | --- | --- |
| Fresh fruit  (data-field 1309) | Pieces (1 apple, 1 banana, 10 grapes, etc) | Apple: 100 g; banana: 100 g; orange: 160 g; pear: 170 g | Apple: 1.3; banana: 0.8; orange: 1.7; pear: 1.6 | 2.0 |  |
| Dried fruit  (data-field 1319) | Pieces (1 apricot, 1 prune, 10 raisins) | Apricot: 8 g; Prune: 10g; Raisins: 10 raisins = 15 g | Prunes: 5.7 g; Raisins: 2.0 g; Apricots: 7.7 g (Fruit and Nuts the first supplement 5th ed) | 0.5 |  |
| Cooked vegetables  (data-field 1289) | Heaped tablespoons | Baked beans: 40 g; peas: 30g; carrots: 40g sweetcorn: 30 g | Baked beans: 3.8 g; peas: 4.0 g; carrots: 2.1 g; sweetcorn: 2.6 g | 1.0 |  |
| Raw vegetables  (data-field 1299) | Heaped tablespoons |  |  | 1.0 |  |
| Bread type  (data-field 1448) Bread intake  (data-field 1438) | Slices | White bread: 36 g | 1.9 | 0.68 |  |
|  |  | Brown bread: 36 g | 3.5 | 1.26 |  |
|  |  | Wholemeal bread: 36 g | 5.0 | 1.80 |  |
|  |  | Other/do not know/prefer not to answer |  | 1.25 |  |
| Breakfast cereal type  (data-field 1468)  Breakfast cereal intake  (data-field 1458) | Bowls | Bran cereal: 40 g | 17.9 | 7.16 |  |
|  |  | Biscuit cereal: 40 g | 7.3 | 2.92 |  |
|  |  | Oat cereal: 160 g | 1.2 | 1.92 |  |
|  |  | Muesli: 55 g | 7.6 | 4.18 |  |
|  |  | Other (e.g. cornflakes): 30 g | 1.8 | 0.54 |  |
|  |  | Do not know/prefer not to answer |  | 3.34 |  |
|  | | | | | |
|  |  |  |  |  |  |
